# Acoustic traits of bat-pollinated flowers compared to flowers of other pollination syndromes and their echo-based classification using convolutional neural networks

**DOI:** 10.1371/journal.pcbi.1009706

**Published:** 2021-12-16

**Authors:** Ralph Simon, Karol Bakunowski, Angel Eduardo Reyes-Vasques, Marco Tschapka, Mirjam Knörnschild, Jan Steckel, Dan Stowell

**Affiliations:** 1 CoSys-Lab, Antwerp University, Antwerp, Belgium; 2 Nuremberg Zoo, Nuremberg, Germany; 3 Machine Listening Lab, Centre for Digital Music (C4DM), Queen Mary University of London, United Kingdom; 4 Florida Spectrum Environmental Services, Fort Lauderdale, Florida, United States of America; 5 Institute for Evolutionary Ecology and Conservation Genomics, University of Ulm, Ulm, Germany; 6 Smithsonian Tropical Research Institute, Balboa, Panama; 7 Museum für Naturkunde, Leibniz-Institute for Evolution and Biodiversity Science, Berlin, Germany; 8 Tilburg University/Naturalis Biodiversity Center, The Netherlands; Tel-Aviv University, ISRAEL

## Abstract

Bat-pollinated flowers have to attract their pollinators in absence of light and therefore some species developed specialized echoic floral parts. These parts are usually concave shaped and act like acoustic retroreflectors making the flowers acoustically conspicuous to the bats. Acoustic plant specializations only have been described for two bat-pollinated species in the Neotropics and one other bat-dependent plant in South East Asia. However, it remains unclear whether other bat-pollinated plant species also show acoustic adaptations. Moreover, acoustic traits have never been compared between bat-pollinated flowers and flowers belonging to other pollination syndromes. To investigate acoustic traits of bat-pollinated flowers we recorded a dataset of 32320 flower echoes, collected from 168 individual flowers belonging to 12 different species. 6 of these species were pollinated by bats and 6 species were pollinated by insects or hummingbirds. We analyzed the spectral target strength of the flowers and trained a convolutional neural network (CNN) on the spectrograms of the flower echoes. We found that bat-pollinated flowers have a significantly higher echo target strength, independent of their size, and differ in their morphology, specifically in the lower variance of their morphological features. We found that a good classification accuracy by our CNN (up to 84%) can be achieved with only one echo/spectrogram to classify the 12 different plant species, both bat-pollinated and otherwise, with bat-pollinated flowers being easier to classify. The higher classification performance of bat-pollinated flowers can be explained by the lower variance of their morphology.

## Author summary

Bats orient and forage by night relying on their biosonar system. Consequently, some plant species that are pollinated by bats evolved echo acoustic signals to attract their nocturnal pollinators. Only few of the more than 400 bat-pollinated neotropical plants have been studied in this regard and it remained unclear if bat-pollinated flowers share general echo acoustic adaptations and differ acoustically from flowers that are pollinated by other animals (e.g. birds, insects). In our study we directly compare the acoustics and morphology of flowers from plants belonging to different pollination syndromes. We compared six species pairs from different families and for each species we collected 14 individual flowers. With an automated ensonification setup we measured the echoes from 202 positions around the opening of each flower. We also took morphological measurements of each flower. We found that the acoustic reflectivity of the flowers, measured as target strength, was higher for bat-pollinated flowers irrespective of their size. To understand if flowers have species-specific acoustic signatures we trained a convolutional neural network to classify flowers by their echoes. We show that a high classification performance can be achieved already with a very limited number of echoes and that bat-pollinated flowers tend to be easier to classify. Our study not only provides new insights into the acoustics of floral signals but also is relevant from a signal processing point of view as we demonstrate how complex objects can be characterized with a very limited amount of acoustic data.

## Introduction

The more relevant information about the environment an animal can extract from the continuous sensory information flow, the better and more efficiently it can navigate, and the more resources it may access [1]. This applies to visual information but also to other perceptual modalities as animals largely benefit from information acquired through all sensory channels. In order to obtain relevant information, the stream of information is filtered, evaluated and compared with known sensory templates. This is commonly referred to as a classification task [2]. Besides classification, it might also be informative to assess how certain variables in the stream of information change over time. In the case of a nectar-feeding bat searching for flowers, this stream of information will not only consist of visual [3] and olfactory information [4] but also of echo acoustic information. The latter is particularly important for detection but also during the approach of flowers [5–8]. It has been debated to which extent plant and flower echoes contain information about the physical form [9,10] and whether echolocating bats can identify and classify plants and flowers by their echoes alone [9,11,12].

Insectivorous bats are able to detect and categorize flying insects echo-acoustically by evaluating small modulations in the amplitude and frequency domain of an echo called “acoustic glints”. These glints are caused by reflections of the impinging sound waves at the fluttering wings of flying insects [13–15]. Similarly, it has been shown for nectar-feeding and omnivorous phyllostomid bats that they can classify and categorize objects of different form independent of their size, which implies that they are capable of acoustic generalizations of objects (*Glossophaga soricina* [16]; *Phyllostomus discolor* [17]. Such capabilities were further confirmed by neurophysiological studies (*Phyllostomus discolor* [18]). Those bats are able to correctly classify different degrees of “impulse response roughness” in the echoes of ultrasound calls reflected by different types of trees (e.g. deciduous trees and conifers; [19]). Impulse response roughness is an echo feature that describes the time/amplitude structure of a reflected signal. A time signal with equally high, closely spaced peaks (as expected from conifers for example) would be regarded as smooth, whereas a signal with peaks of different height and larger spaced peaks (as expected from deciduous trees) is coarse. Moreover, echo-acoustic measurements of trees and shrubs show that their echoes are complex and highly variable [12]. Echo-acoustic classification based on the magnitude of the echoes’ spectrograms, a feature obviously perceived by bats, are thus likely to permit species-specific assignment [11]. Being able to acoustically recognize and categorize plant species might be beneficial for insectivorous bats as they can be used as landmarks [20] and/or to select feeding sites. For nectarivorous and frugivorous bats, however, recognizing plants by their echoes is of paramount importance as they are their primary food source. There is some evidence that characteristic differences in flower morphology lead to distinctive echoes [9] which could be recognized by bats. Moreover, for two plant species it was shown that they have specialized reflective floral parts which make the flowers acoustically conspicuous and facilitate detection [5,7]. Nectar-feeding bats foraging on these flowers depend on these signals and have difficulties to find flowers when these signals are missing [5]. This raises the question why there are not more bat-pollinated plant species that developed floral acoustic adaptations. Moreover, how do echo features of bat-pollinated flowers compare to echo features of flowers belonging to other pollination syndromes such as entomophilous (insect-pollinated) or ornithophilous (bird-pollinated) flowers?

To approach these questions, we conducted a series of morphological and echo acoustic measurements of 168 flowers from 12 different neotropical plant species belonging to 6 different families. For each family we chose a bat-pollinated and a hummingbird- or insect- pollinated plant that were both flowering sympatrically. We acquired echo recordings of these flowers, from different angles, and analyzed their spectral target strength for different frequency bands. We trained a deep learning classifier (a convolutional neural network, CNN) with echo spectrograms of the flowers and classified a test set of flower echoes. We predicted that echo-acoustic properties (such as target strength) of bat-pollinated flowers differ from those of flowers belonging to other pollination syndromes and that bat-pollinated flowers are easier to classify.

## Results

**Fig 1:**
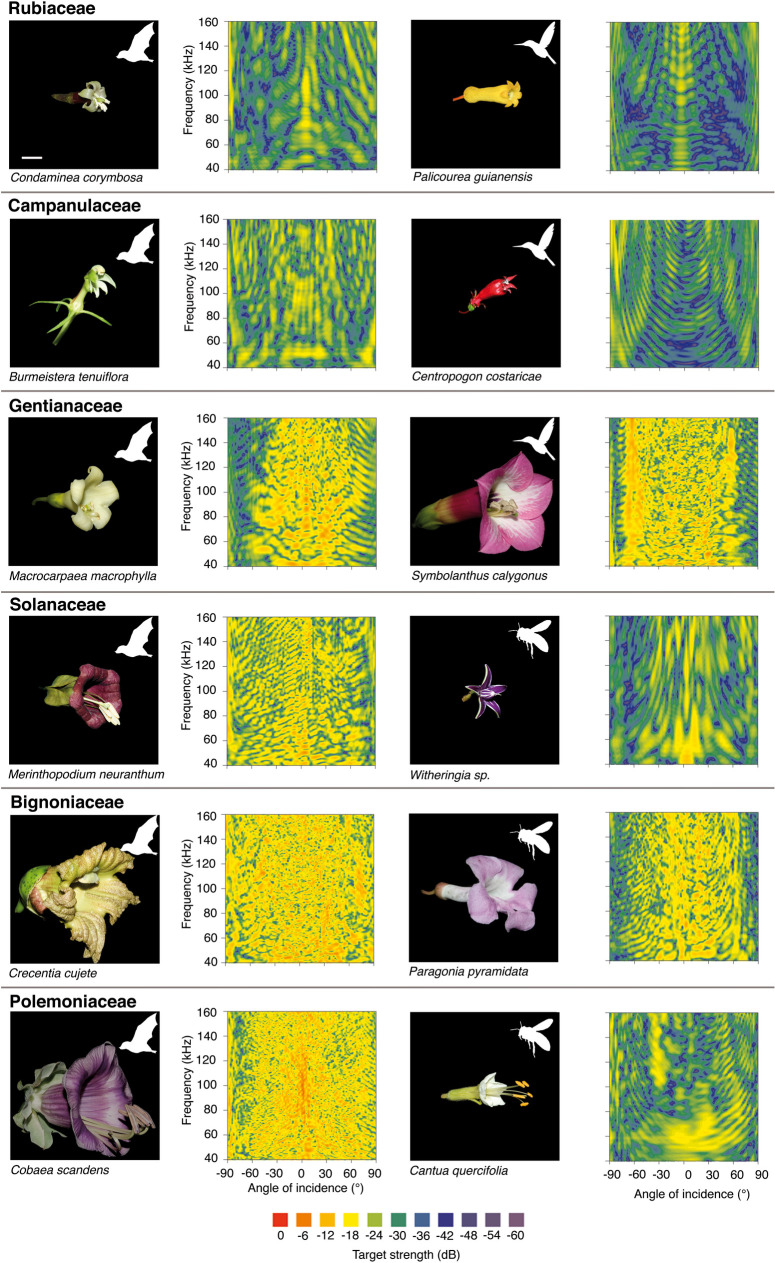
Images and directional spectra of flowers from 6 different plant families. As a visual representation of the flowers’ echo characteristics, the echo spectra were plotted over the angle of sound incidence, which resulted in spectral directional plots. The angle of sound incidence represented the position from which the echo was acquired, 0° represents the opening of the flower, 90° represents the side, images were taken from 45°. The target strength, or echo intensity, is given in a colour gradation (see colour bar below). The images and directional spectra on the left are from the chiropterophilous plant species, at the right, the flowers are from insect- or bird-pollinated species, as indicated by the white symbols in the images. The bar in the upper left image represents 10 mm, all flower images are on the same scale.

**Fig 2:**
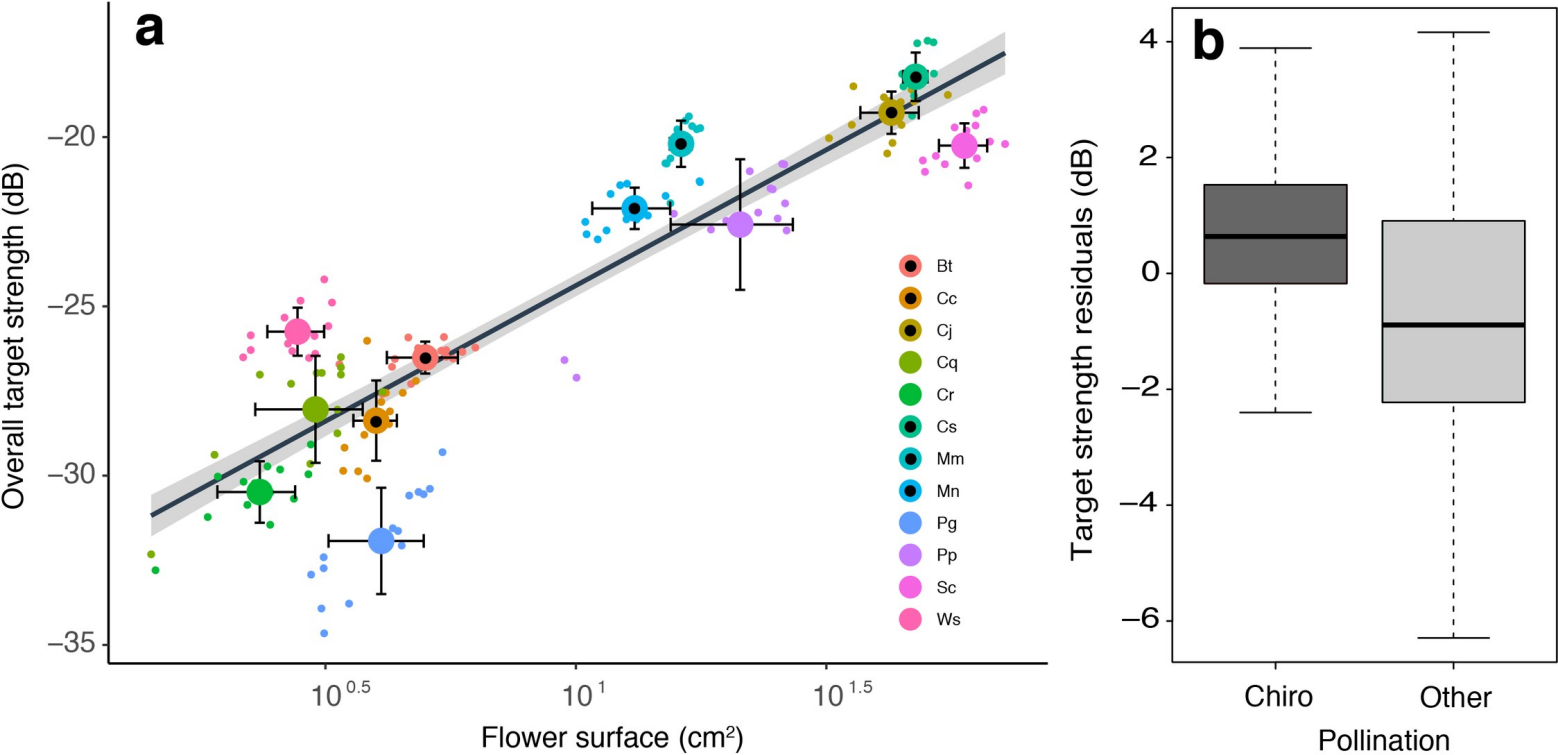
Overall target strength of flowers compared to their surface area and pollination syndrome. (**a**) Dependence of the overall spectral target strength of a broad frequency band (40–160 kHz) on the flower surface area. Bat-pollinated species are indicated with circles with black filling. Bt = *Burmeistera tenuiflora*, Cc = *Condaminea corymbosa*, Cj = *Crescentia cujete*, Cq = *Cantua quercifolia*, Cc = *Centropogon costaricae*, Cs = *Cobaea scandens*, Mm = *Macrocarpea macrophylla*, Mn = *Merinthopodium neuranthum*, Pg = *Palicourea guanensis*, Pp = *Paragonia pyramidata*, Sc = *Symbolanthus calygonus*, Ws = *Witheringia* sp. (**b**) Residuals in target strength from the estimated linear model (see regression line in A) for flowers grouped by their pollination syndrome. The category ‘other’ contains insect- and bird-pollinated flowers.

The flowers we investigated differed considerably in size and form and consequently showed differences in their spectral directional characteristics (Fig 1). Bat-pollinated flowers did not share a common spectral signature or a feature that was different from that of flowers not pollinated by bats. We took different morphological measurements of each of the flowers such as the length of the corolla, the length of the calyx, and the inner as well as outer diameter of the flowers opening in the horizontal and vertical plane. With these measurements, we estimated the surface area of the flowers and compared them to the average target strength of the directional spectra. The surface area of the flowers had an influence on overall target strength, TS (40 kHz—160 kHz). Flowers with larger surface area had a higher mean target strength (y = 3.488 ln (x) - 32.422; F_1,166_ 656.6; R^2^ = 0.797; p < 0.0001; Fig 2A). A linear mixed effect model including an interaction between surface and pollination explained more of the variance in overall target strength than a model with only surface as fixed factor (LMM plant family as random factor, χ^2^(2) = 113.29, *p* < 0.0001). After removing the effects of flower surface area, bat-pollinated flowers had a significantly higher overall TS than insect- and bird-pollinated flowers (Independent samples T-test with residuals: t = 4.716, df = 122.64; p < 0.0001, Fig 2B). The influence of the surface area on the overall target strength was also evident when different frequency bands were analysed separately (see S1–S4 Figs). Interestingly, the overall TS values for all flowers of *Macrocarpea macrophylla* for the highest frequency band formed a cluster and had values well above the predicted values for this flower size (average residual 5.0 dB ± 0.6).

In addition to size, flower shape had a major influence on the directional spectra. Similar-shaped flowers, whether on the species or the individual level, had similar directional spectra. For example, all bell-shaped flowers (*Macrocarpaea macrophylla*, *Symbolanthus calygonus*, *Merinthopodium neuranthum*, *Crecentia cujete*, *Paragonia pyramidata* and *Cobaea scandens*) shared similar patterns in their directional spectra (Fig 1). They were characterized by frequent occurrence of interference patterns, i.e. reinforced frequencies in close vicinity to cancelled frequencies. The positions of these interference patterns within the spectrograms varied strongly, depending on the angle of sound incidence. This, in turn, resulted in a “spotted” pattern of the directional spectral plot. Moreover, it revealed that there is a relation between the size of the bell-shaped flowers and the occurrence of interferences (left column of Fig 1, Gentianaceae, Solanaceae, Bignoniaceae, Polemoniaceae; flowers in this column are arranged in from smallest to largest). The larger the flower, the more interferences occurred. This led to a more blurred or noisier appearance of the directional spectral plots.

### Morphological flower features

For all individual flowers we deduced morphological features, such as length of the corolla and calyx, depth, and inner and outer diameter of the corolla in the horizontal as well as in vertical plane. As the flowers from different species differed considerably in size, we chose the coefficient of variation (*c*_*V*_) for an interspecific comparison of variability for different morphological features. *c*_*V*_ is the ratio of standard deviation (*σ*) to the mean (*μ*), and hence is dimensionless.


$$c_{V}=\frac{\sigma }{\mu }$$ (1)

**Fig 3:**
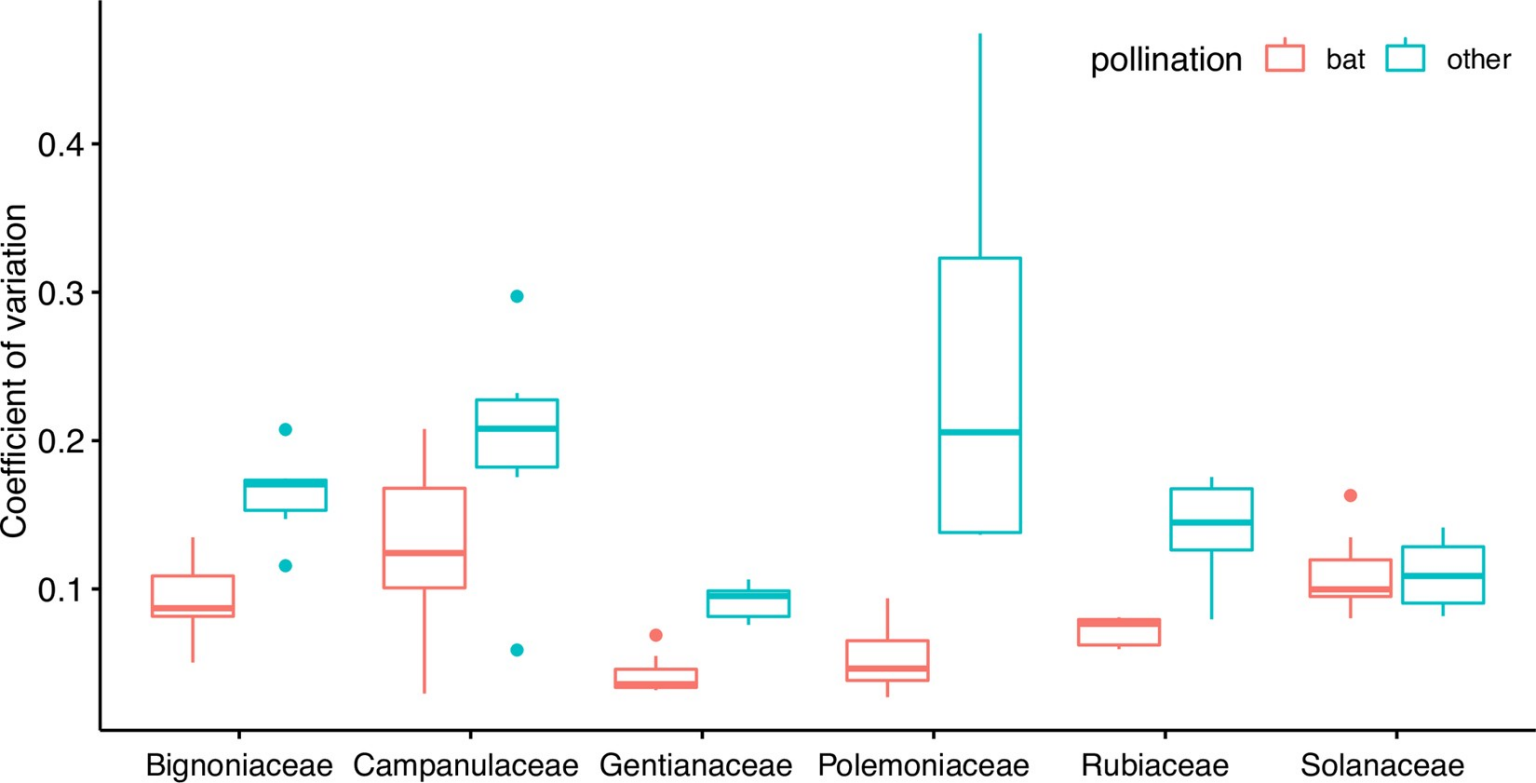
Coefficients of variation for the different morphological flower features for twelve plant species from six different families. Orange: bat-pollinated species. Petrol: bird-or insect-pollinated species. For each species, the coefficients of variation of the length of the corolla, the length of the calyx, and the inner as well as outer diameter of the flowers opening (in the horizontal and vertical plane) was calculated.

To quantify the morphological variation within the 14 flowers we collected for every species, we determined the *c*_*V*_ for each of the morphological features, such as length of corolla, length of calyx, depth, and inner as well as outer diameter of the flowers opening in the horizontal and vertical plane. In all taxa, coefficients of variation were always higher for insect- and bird-pollinated species than for bat-pollinated species (Fig 3).

The *c*_*V*_ of bat-pollinated flowers was significantly lower than the *c*_*V*_ of insect- and bird-pollinated species (Linear Mixed Model [LMM] with pollination syndrome as fixed factor and plant family as random factor, χ^2^(1) = 24.085, p < 0.0001). The mean *c*_*V*_ of bat-pollinated flowers was 0.08 whereas flower morphology of insect- and bird-pollinated species was more variable, as revealed by a *c*_*V*_ of 0.16.

### Classification with a convolutional neural network

**Fig 4:**
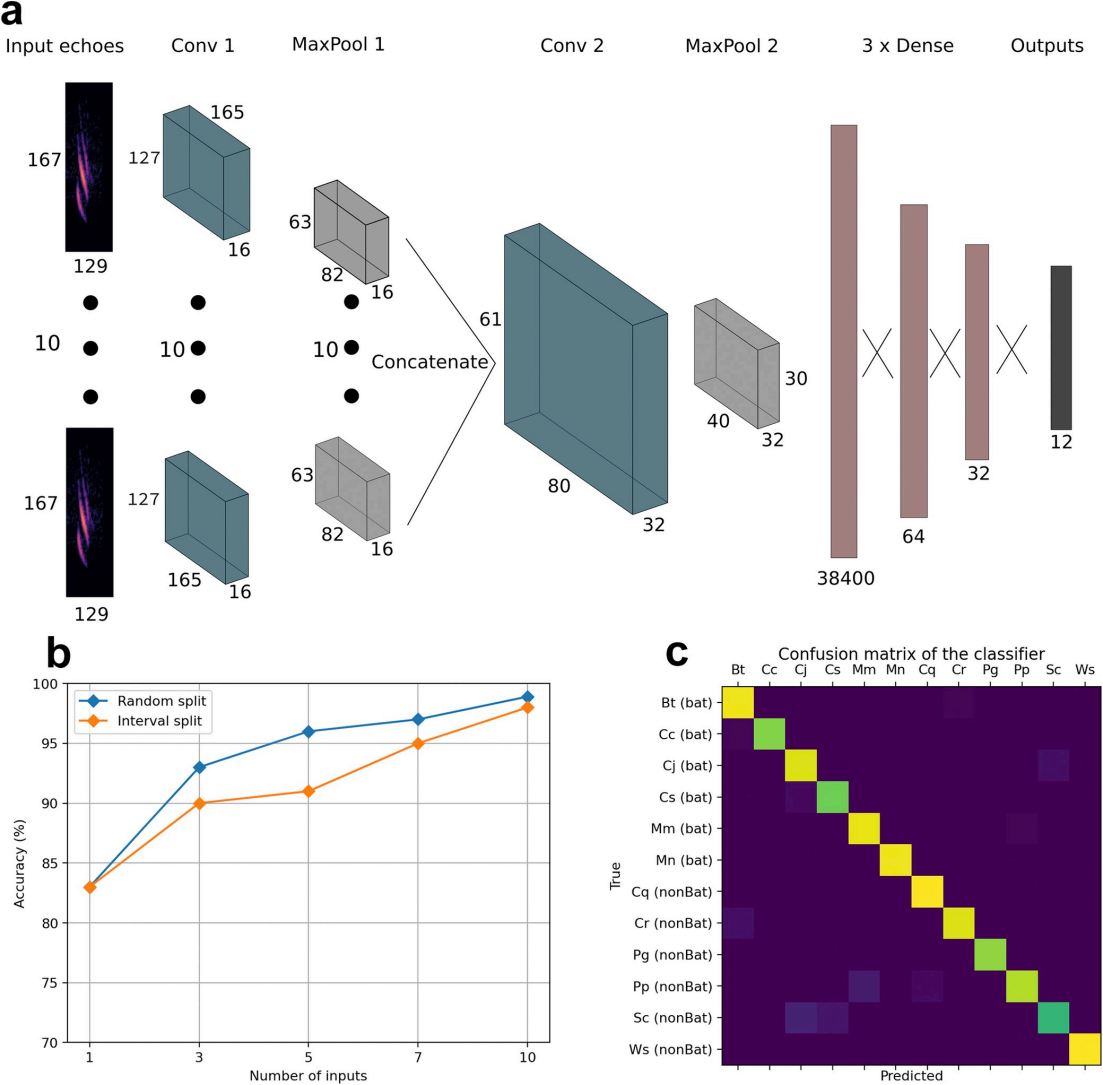
Echo classification using convolutional neural networks. (**a**) Architecture of the neural network. (**b**) Results of the CNN model on two different iterations of the data and different amounts of echoes as the input. (**c**) The confusion matrix of a trained 10-input CNN on the testing set.

Our CNN classifier was evaluated on a testing set derived from individual flowers which had not been used at any point in time during training (Fig 4A). In all cases models trained on echoes sampled randomly performed better than respective implementations trained with the interval sampling, especially when classifying between 3 to 7 echoes, with 10 echoes the difference being negligible. The CNN was generally able to distinguish between any of the 12 classes well. Across the different number of inputs classification performance of plant species based on the echoes was between 84 and 98%. There was a positive correlation between the number of inputs and classification accuracy, with the largest gain when increasing the number of echoes from 1 to 3. When the input to the CNN was just a single echo, the classification accuracy was 84%, and rose as high as 98% with 10 echoes (Fig 4B).

The classifier’s confusion matrix (Fig 4C) showed some compelling characteristics. The convolutional neural network performed considerably better in recognizing classes representing species pollinated by bats, than those pollinated by insects and hummingbirds, and this difference showed especially in earlier stages of training. When evaluated on 10 echoes the species classification accuracy for bat and non-bat-pollinated flowers was as follows: After 10 epochs 73% for bat-pollinated and 51% for non-bat-pollinated (95% confidence intervals, using binomial method for classification correct detection rate [21]: 69.3–77.5% versus 46.9–55.9%). After 40 epochs 97% for bat-pollinated and 93% for non-bat-pollinated (confidence intervals 95.8–99.0% versus 91.1–95.7%). The network’s convergence depends on amount of inputs and takes longer the more inputs are processed simultaneously. With 1–5 inputs, the network achieved good results only after approximately 20 epochs, and around 40 epochs with 10 inputs.

## Discussion

Nectar-feeding bats have to find and revisit hundreds of flowers every night to fulfil their energetic needs. Species-specific acoustic information may help them to decide which flowers they should exploit. It will facilitate recognition of suitable, bat-pollinated and thus nightly nectar secreting flowers. Moreover, species-specific echo sequences will inform the bats about flower morphology and how to get to the nectar. Therefore, acoustic information on floral shape, in addition to the olfactory and visual cues, provides crucial support for efficient foraging.

### How much information do nectar-feeding bats acquire during their approach?

Nectarivorous and frugivorous phyllostomid bats emit echolocation signals during search flight at a rate of 15 ± 5 calls per second, which increases up to 32 ± 11 calls per second when approaching a target [22,23]. Our own observations with the species *Glossophaga soricina* showed that during behavioural experiments, when bats had to accomplish echo-acoustic discrimination tasks, repetition rate increases to up to 51 calls per second. As flying bats continuously change their position between echolocation call emissions they will receive target echoes from many different directions. Moreover, bats are known to investigate flowers of interest by hovering briefly in front of them [6]. They may actively acquire important echo information from the respective object and are likely to perform an “auditory scene analysis” where echo information is integrated over several calls [24]. Thus, spectral information similar to the information of the directional spectra, which consisted of 101 single spectra acquired from different directions (Fig 1), should be available to the bats within 2 to 3 seconds of an approach flight assuming repetition rates of 32 to 51 calls per second.

Such an “auditory scene analysis” can be based on different echo features, but is a spectrogram based auditory scene, like we assumed it for our CNN, realistic? Bats are able to deduce many aspects of the impulse response from the echoes and thus can use highly resolved temporal information i.e. range profile information [25]. Bats can clearly perceive spectral echo information [26], and they are known to use both spectral and temporal information [27,28] and may have spectrogram-like representations [10]. This is supported by many target discrimination tasks where spectral echo information was found to be a decisive cue for bats [16,26,29–33]. Moreover, our results suggest that spectral echo sequences acquired from different directions are particularly informative, and even single flower echoes or small sequences contain species-specific information.

### Are echoes of bat-pollinated flowers louder?

After removing effects of flower surface area, we found a higher overall target strength for bat-pollinated flowers than for flowers pollinated by insects or birds. The maximum distance at which an object can be detected echo acoustically by a bat depends on the target strength of the object [34]. Increased target strength of bat-pollinated flowers will increase echo acoustic detection distance. Furthermore, separation of target echoes and clutter echoes should also be favoured when the target echo is of higher intensity. What could be the reason for the increased target strength of bat-pollinated flowers? Sound waves are reflected at the border of two media, for example at the border between water and air. In case of flowers it would be the border of air and the waxy cuticle of the flowers’ petals. Sound waves will be mainly reflected when the two media have different acoustic impedances [35,36] and will enter the medium if the acoustic impedances are equal or similar. The greater the difference in acoustic impedance of the two media, the greater is the magnitude of the reflected echo [35]. The acoustic impedance is determined by the product of speed of sound and the density of the medium, thus the magnitude of the reflected echo (i.e. target strength) increases when density of the medium increases. The stiff and waxy surfaces of many bat-pollinated flowers [37,38] might be an adaptation to increase density and thereby their acoustic impedance with respect to the acoustic impedance of air. This would be a mechanism to enhance flowers’ target strength and increase their echo acoustic detection distance.

Bat-pollinated flowers show less variance in their morphological features than entomophilous or ornithophilous flowers. Although the present study, with a total of 32320 flower echoes, from 168 individual flowers, is one of the most extensive studies on echoacoustic adaptions of flowers, the amount of species covered is still rather limited (n = 12). However, as we consistently found a more conserved flower morphology and a better classification performance by our CNN for bat-pollinated flowers, we suggest that the conserved flower morphology is an acoustic adaption. Conserved morphology will lead to less variant echo characteristics, which in turn favours classification performance. To test if the lower variance in morphological features is directly resembled in a lower variability of echo features we determined the coefficient of variation (*c*_*V*_) for the target strength (TS) values of the different frequency bands.

**Fig 5:**
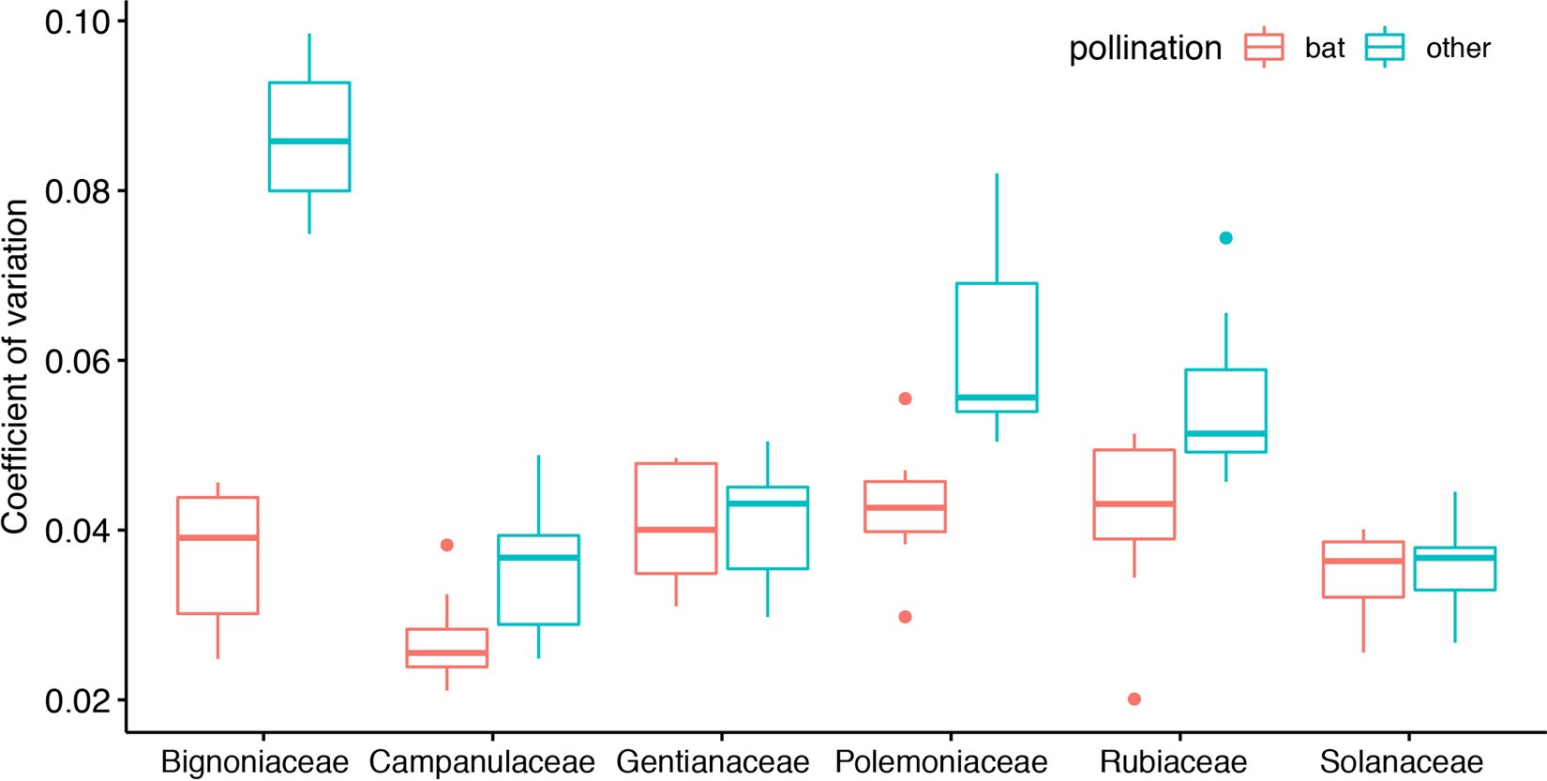
Coefficients of variation of acoustic features of the echoes of flowers from twelve plant species from six different families. Orange: bat-pollinated species. Petrol: bird-or insect-pollinated species. For each species, we analysed the variation of the average target strength of four frequency bands for two scans (each with 101 measurements). We analysed a 45 kHz, 68 kHz, 102 kHz and a 153 kHz frequency band. Upper and lower boundary of the bands where ±20% around these centre frequencies.

We found that the *c*_*V*_ of the TS of bat-pollinated flowers was significantly lower than the *c*_*V*_ of the TS of insect- and bird-pollinated species (Linear Mixed Model [LMM] with pollination syndrome as fixed factor and plant family as random factor, χ^2^(1) = 33.821, p < 0.0001, Fig 5). This indicates that a high morphological variance is directly related to higher acoustic variance. An explanation could be that bird- or insect-pollinated flowers are less constrained regarding their morphology as they lure their pollinators by conspicuous colours and slight differences in morphology will not influence their detectability. For bat-pollinated flowers the morphological fit between pollinator and flower is more important, moreover, the lower morphological variance facilitates echo acoustic detection.

Our deep learning study is in a similar spirit as prior work which used machine learning to classify objects based on their acoustic reflections. Support vector machines (SVM) have been used to classify plants [11], and multi-layer perceptron (MLP) to classify Platonic shapes [39]. In these prior works, the acoustic excitations were bat-inspired but synthetic frequency sweeps, whereas we used recorded bat echolocation calls (synthetically, via impulse responses). Perhaps more importantly, our deep learning method has a particular advantage over the machine learning algorithms previously used: a CNN can use a high-dimensional “image” such as an echolocation spectrogram as direct input to the algorithm, and does not require the data to be preprocessed by feature extraction or dimension reduction. The machine learning algorithm is thus able to make use of subtle discriminative cues in the signal which might otherwise be discarded. Further, we designed a custom CNN architecture that integrates the information from multiple echoes processed in parallel, allowing that the information can be combined holistically and non-linearly to produce the final decision. In these ways, our machine learning method moves closer to being a proxy for the type of inferences that an echolocating bat may be making. Moreover, our method might be an approach to classify artificial shapes and objects. Acoustic reflector shapes inspired by floral reflectors have already been shown to be useful as sonar landmarks for autonomous navigation [40]. Therefore, our approach might also be relevant for autonomous systems which are equipped with technical sonar systems.

Our CNN is not directly modelled on biological neural organisation, and the analogy is still highly heuristic. In particular, our CNN input is simply a set of acoustic echo spectrograms, presented in parallel, without contextual information such as (relative) angle. Bats do not emit 10 calls at once but in sequence, and may produce/analyse them dependently on each other: previous echoes can influence the interpretation of the current ones. Bats also presumably make use of timing and (relative) angle of echoes in their decisions. These observations could perhaps facilitate future work in implementing an ‘angle aware’ network, which, in addition to receiving an echo, would receive the angle from which this echo comes from and process the echoes sequentially (e.g. via a recurrent neural network). The production of further calls, or even the next choice of observation angle, could depend on the previous measurements. This online problem-solving setting would then be of the kind studied in reinforcement learning. These avenues offer future ways to study echolocation as an information-gathering strategy, by using machine learning to emulate real-time echolocation behaviour.

## Materials and methods

### Plant species

A total of 168 flowers from six families and twelve species were collected and measured. For each family, we collected 14 flowers of a bat-pollinated species and 14 flowers of a species pollinated by insects or birds and we tried to collect flowers not only from one individual plant but from at least 3 and up to 14 different plants (Table 1). With the exception of the species pair belonging to the family Polemoniaceae, all pairs of species occurred and flowered syntopically (in the same habitat). The Polemoniaceae pair flowered sympatrically (in the same geographic region) around 100 km apart. Flowers were collected in the field, kept fresh with wet cellulose tissue and brought to the laboratory at nearby field stations for immediate measurement. Studies were conducted at two locations in the mountain rainforest of south-east Ecuador at the Estación Científica San Francisco (ECSF) situated at 1820 m, halfway between Loja and Zamora (Longitude: 79° 4’41.56”W; Latitude: 3°58’22.52”S) and in the lowland rainforest of central Costa Rica at La Selva Biological Station (Organization for Tropical Studies; Longitude: 84° 0’12.92”W; Latitude: 10°25’52.61”S). All experiments were approved by the local authorities (Ecuador: Ministeria del Ambiente, autorizacion para investigación científica N. 035-DPA-MA-2012; Costa Rica: Ministerio del Ambiente y Energia, RESOLUCIÓN No 102-2009-SINAC).

**Table 1:** Plant families, species, number of individual plants the flowers were collected from and the corresponding pollinators described in the literature. For each of the species we collected 14 individual flowers.

Family	Species	Number of plants	Pollinator	Literature
Bignoniaceae	*Crescentia cujete*	4	Chiroptera	[37]
Bignoniaceae	*Paragonia pyramidata*	3	Insecta/*Xylocopa* sp.	[41]
Campanulaceae	*Burmeistera tenuiflora*	10	Chiroptera	[42]
Campanulaceae	*Centropogon costaricae*	9	Trochilidae	[43]
Gentianaceae	*Macrocarpea macrophylla*	5	Chiroptera	[44]
Gentianaceae	*Symbolanthus calygonus*	14	Trochilidae	[45]
Polemoniaceae	*Cobaea scandens*	7	Chiroptera	[38]
Polemoniaceae	*Cantua quercifolia*	5	Sphingidae	[46]
Rubiaceae	*Condaminea corymbosa*	10	Chiroptera	[38]
Rubiaceae	*Palicourea guianensis*	4	Trochilidae	[47]
Solanaceae	*Merinthopodium neuranthum*	8	Chiroptera	[48]
Solanaceae	*Witheringia* sp.	4	Insecta	[49]

### Echo measurements

**Fig 6:**
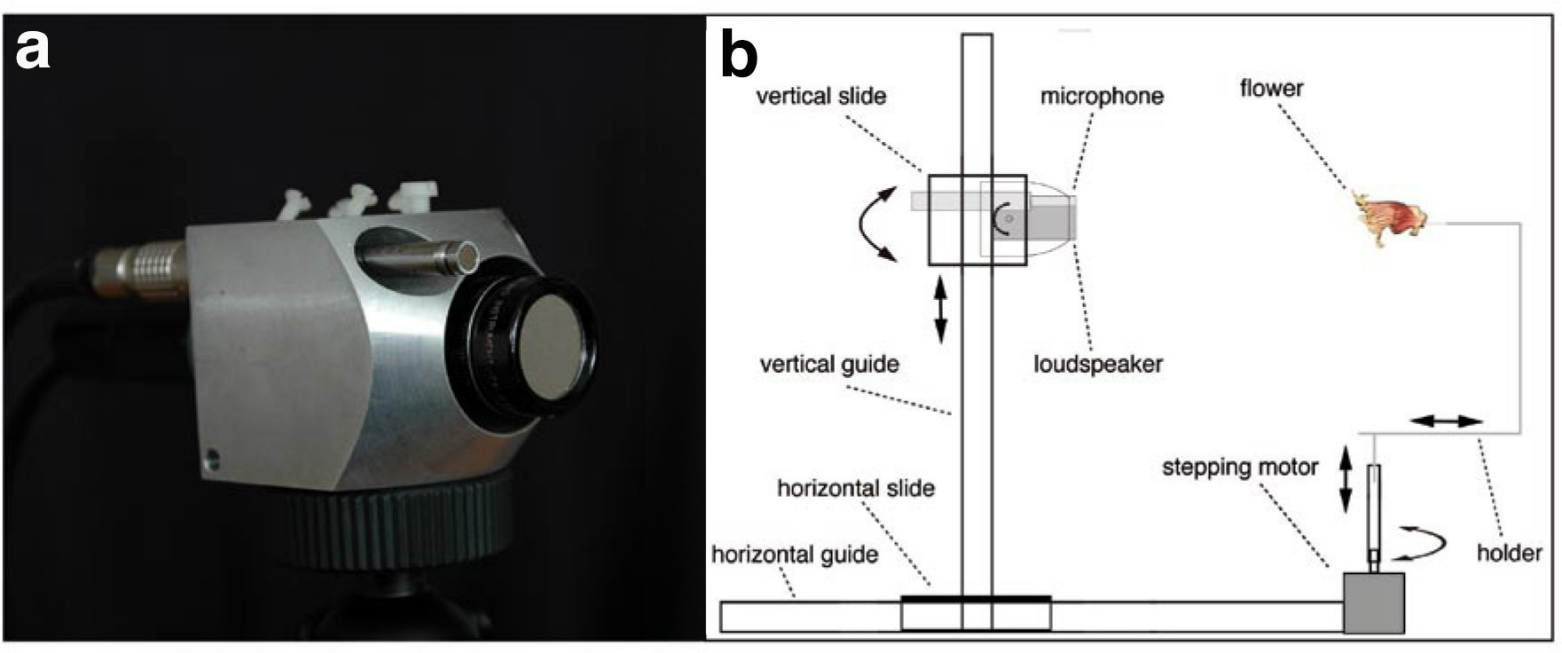
The setup used to measure ultrasonic echoes. (a) The biomimetic sonar head with the ¼” G.R.A.S. microphone and the custom build EMFI- transducer. (**b**) Schematic illustration of the measuring setup.

The setup to measure ultrasonic echoes reflected from targets, here flowers, consisted of two main components: the biomimetic sonar head (Fig 6A) and the unit where objects could be fixed and rotated. Both were installed on aluminum guides made out of aluminum profiles (item Industrietechnik GmbH, Solingen, Germany). To turn the objects and ensonify them from different directions, we used a high precision stepping motor (Faulhaber GmbH, Schönaich, Germany). We used a U-formed metal frame, which allowed fixing the objects from their hind side and freely adjusting the center of rotation. The high precision stepping motor was controlled by a computer and turned the flowers in 1.8° steps. The biomimetic sonar head (representing a “bat head”) consisted of a loudspeaker and microphone, which were installed in a solid aluminum body on a vertical guide (Fig 6B). To mimic the movement of a bat’s head during approaches to a target, the aluminum body had two fixtures for a microphone to the left and right (45°) of the centrally located loudspeaker at a distance of 25 mm between the centre of the microphone and the centre of the loudspeaker (Fig 6A). For most of the measurements, we only used the right microphone which was a ¼” free-field microphone type 40BF in combination with the preamplifier 26AB connected to the power module 12AA (all from G.R.A.S. Sound & Vibration, Holte, Denmark). We used a custom-built EMFit (Electro Mechanical Film) transducer as loudspeaker. The EMFit—transducer and the appropriate amplifier were built in cooperation with the Department of Sensor Technology at the Friedrich-Alexander University Erlangen-Nuremberg, Germany. The transducer had a membrane diameter of 16 mm and was supplied with a voltage of U = 600 Vpp. This resulted in sound pressure levels of approximately 80 ± 6 dB (frequency range: 40 kHz to 160 kHz) at a distance of 1 m. Loudspeaker and microphone were connected to a multifunction data acquisition (DAQ) device Ni PCIe-6251 (16 bit, up to 1,25 MS/s) through a BNC terminal BNC-211 0 (both from National Instruments, Austin, Texas). The DAQ device was integrated into a custom built Mini-ITX PC (MoDT Flex 945, AOpen, Taipeh, Taiwan) and was controlled by the program ECHOlab version 1.0–4.3 (written in LabVIEW by Ralph Simon and Benjamin Fink). The whole setup was constructed for mobile use during field trips and was run with 12 V or 110–240 V, respectively.

For the measurements the distance between the front edge of the blossom and the front edge of the transducer was adjusted to 20 cm. Flowers were fixed at their back with a thin metal needle. This needle was mounted on the U-shaped holder (see Fig 6B), which was fixed on the extended axis of a stepper motor that permitted exact and reproducible rotation with defined increments. As the U-shaped holder was adjustable, the center of rotation for the objects could be varied. We always chose the front edge of the flowers’ corolla as center of rotation so that the sound propagation direction was perpendicular to the plane of flower opening (0°).

We ensonified the flowers with a constantly repeated MLS (maximum length sequence), which is a periodic pseudorandom binary sequence of zeros and ones (for example: 0001101001110…). It is basically a predetermined noise signal and an important property of any MLS is that its autocorrelation is a very short impulse (Dirac impulse). This means that if the recorded echo is combined with the original MLS in a deconvolution process (similar to a cross-correlation), the impulse response (IR) from the object is obtained. The impulse response can be thought of as an echo of a very short, broadband click and it fully characterizes the transmission properties of any linear and time-invariant (LTI) system. Actual echoes of bat echolocation calls can be derived from the impulse responses by convolution with calls. The advantage of using MLS instead of short impulses is that MLS has a longer duration and thus contains more energy. This results in a much better signal-to-noise ratio (for more information regarding MLS method see Cho, 1990). We used a MLS containing 16 383 samples, which was continuously played at a sampling rate of 500 kHz resulting in a duration of 33 ms. The analogous microphone signal was digitized with a sampling rate of 500 kHz at 16 bit resolution.

Acoustic measurements or scans were taken over a 180° angle from -90° to 90° with regard to the center of the corolla in 1.8° increments. This led to 101 measurements per flower. We scanned most the 168 flowers both in the horizontal as well as in the vertical plane. Thus, we received 202 individual echoes (to recover 202 impulse responses) for each flower acquired from different directions. 15 individual flowers from various species were only measured in one plane, or measurements had to be discarded respectively because the measurement was interrupted mainly due to an overheated amplifier. Replay and recording were sample synchronous. We made sound recordings of the MLS-signal which was backscattered at the objects surface. The raw recordings had a duration of two seconds each and we deduced the impulse response (IR) by deconvolution with the original MLS as described above. As the sequence length of the MLS was 33 ms the 2 s recording consisted 60 identical IRs. To further increase the signal to noise ratio of the IRs we averaged over 50 sequences. In this averaged IRs we searched for the peaks originated from the flowers, we cut them out (rectangular window, 1024 samples) and calculated the power spectral density.

To obtain spectra independent of the frequency response of the loudspeaker, an additional measurement was performed for calibration. We installed a 25 cm x 25 cm plexiglass plate in place of the flowers and measured its echo. To obtain the flowers’ spectral characteristics regardless of the frequency response of the loudspeaker we used this ‘plate measurement’ to calculate the difference between its spectrum and the spectrum of the recorded flower. This permitted us to assess the flowers`spectral target strength. The values we obtained with our method resemble the spectral target strength (TS) at a reference distance of 10 cm. We analyzed the spectral target strength for 5 different frequency bands. One frequency band (40 kHz– 160 kHz) was very broad, thus covering the typical bandwidth of a glossophagine bat’s echolocation call. The others had a lower bandwidth: we choose 45 kHz, 68 kHz, 102 kHz and 153 kHz as center frequencies, upper and lower boundary of the bands where ±20% around these center frequencies. To determine TS values for each flower for these frequency bands, we averaged TS (after delog) for the azimuth and also for the elevation measurements (each consisted of 101 measurements) and then used the mean of azimuth and elevation for statistical comparisons between flower types.

**Fig 7:**
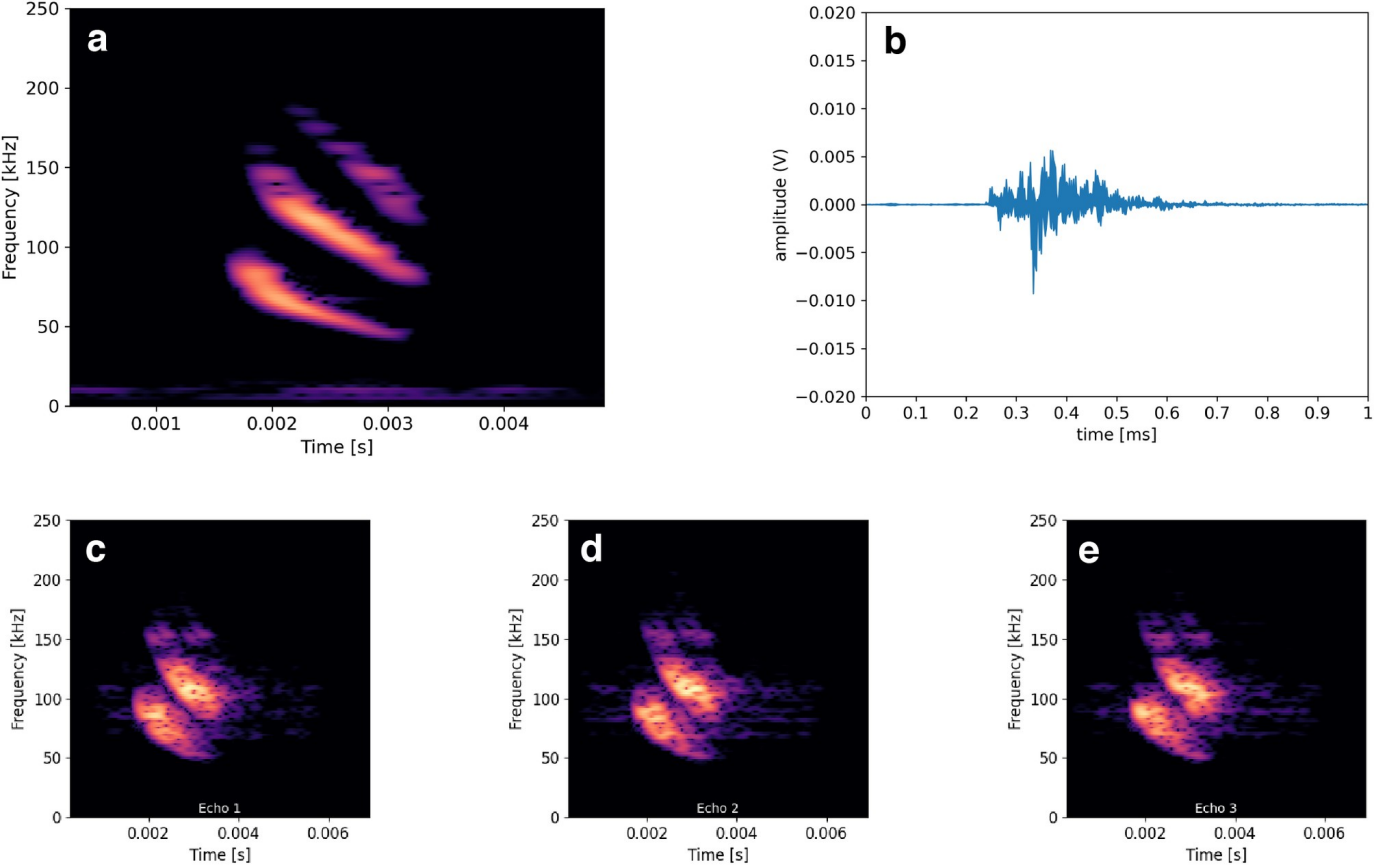
Example spectrograms used as input to the convolutional neural network. (**a**) Echolocation call from Pallas’s long-tongued bat (*Glossophaga soricina*). (**b**) Example impulse response of a flower recording. (**c-e**) Example spectrogram sequence used as input.

Classification with a convolutional neural network (CNN): For each species we had 14 distinct scans of different individual flowers. The distinct scans of each class were separated into training, validation and testing subsets, stratified per individual flower to avoid the risk of overestimating classification performance due to any “overfitting” in the training process. The validation and testing set covered all 12 species, but contained echoes only from individual flowers that had not been used for training. Two instances of each class were put in the test set, eight instances in the train and two in the validation set, equivalent to a split of approximately 58% / 21% / 21%. We derived flower echoes by convolution of a typical echolocation call of *Glossophaga soricina* with the impulse responses we measured from the different angles (see Fig 7). The call was filtered prior to the convolution with a high pass filter with a cutoff of 5kHz, to ensure that no low frequency noise leakage was present. The spectrograms of each echo were calculated with a Hann window of size of 256 samples and a hop size of 90%. This allowed for a good trade-off between time and frequency resolution for this task [50].

Our classifier would use a small number of synthetic echoes to make a prediction, designed as a simplified analogy of a bat using multiple calls on approach to a flower. Only the echo spectrograms were used as input, without any information on the chosen angles or their relation. We studied groups of 1, 3, 5, 7 and 10 echoes. Further, for multi-echo situations we evaluated two ways to select groups of echoes, following different assumptions about a bat’s physical approach.

1) Random split (assumption: bats randomly receive echoes from different positions during approach): Impulse responses from each single instance of all flowers were randomly shuffled along the angle axis, and separated into sets of 3, 5, 7 and 10. This procedure resulted in 4 different sets—each containing all available data in chunks of respectively 3, 5, 7, and 10.

2) Interval split (assumption: bats approach in a narrow trajectory and receive echoes from certain consecutive sequences): Again impulse responses were taken from each instance in chunks of X = (3; 5; 7; 10). This time however, the IRs were selected in structured sequences. A random interval *n* between 2 and 8 was selected and every *n*th impulse response was picked—up to length X. This allowed for a more realistic approximation of an actual bat’s approach, which would include multiple echoes, coming in succession from one direction. Note that this option might result in less diversity within a chunk (increased redundancy of information between echoes) and thus is expected to reduce recognition performance.

The architecture of the CNN is inspired by experiments on classification of plant appearance qualities for flower grading [51]. 1, 3, 5, 7 or 10 echo spectrograms together constitute one simultaneous input sample to the network. Independently of the number of inputs, each echo goes through a ‘preprocessing layer’—consisting of a convolutional layer with 16 channels and a max pooling layer, in which the weights are shared, i.e. the same preprocessing for each spectrogram in the chunk. The results of this preprocessing are then concatenated into one tensor and processed further through another set of convolutional (32 channels) and max pooling layers. Then, three dense layers, and finally output of probabilities for each of the 12 classes. Each convolutional layer has a kernel size of 3x3, with stride 1, and each max pooling layer a kernel of size 3x3 with stride 2. A detailed overview of the architecture can be seen in Fig 3. Note that one scan of one flower in the dataset had been mis-identified during manual labelling: it was labelled as Cc_e_01 but should have been Cs_e_10 (both bat-pollinated). The data of this scan has been removed from plots and analysis. It was included in training of the classifier but not in testing; hence classification results are not over-estimated.

We followed standard good practice to train a CNN deep learning classifier, as follows. Cross-entropy was used as the loss function to measure model performance during training. The Adam optimizer [52] was used for training, with a learning rate of .0001, and batch size of 16. During each epoch training was done over the entire train split in shuffled batches, and then the model was run with validation data, in not shuffled batches, to monitor performance. The final evaluation was done over the test set, after training was finished. Completion of training was decided by early stopping, as well as manual inspection of loss values for train and validation splits. Early stopping is a technique that prevents overfitting by stopping the training process if performance on the train set improves, but performance on the validation set stagnates, or degrades, over a predefined amount of epochs. The creation of an echo spectrogram occurred on-demand in the deep learning system, by convolving a selected impulse response with a bat call and then calculating the resulting spectrogram. This approach slows down processing to some degree, since the spectrograms are not pre-calculated, but allows for quick and convenient changes to the window/hop sizes during training and hyperparameter tuning. An example input to the CNN can be seen on Fig 6C–6E.

### Morphological data

**Fig 8:**
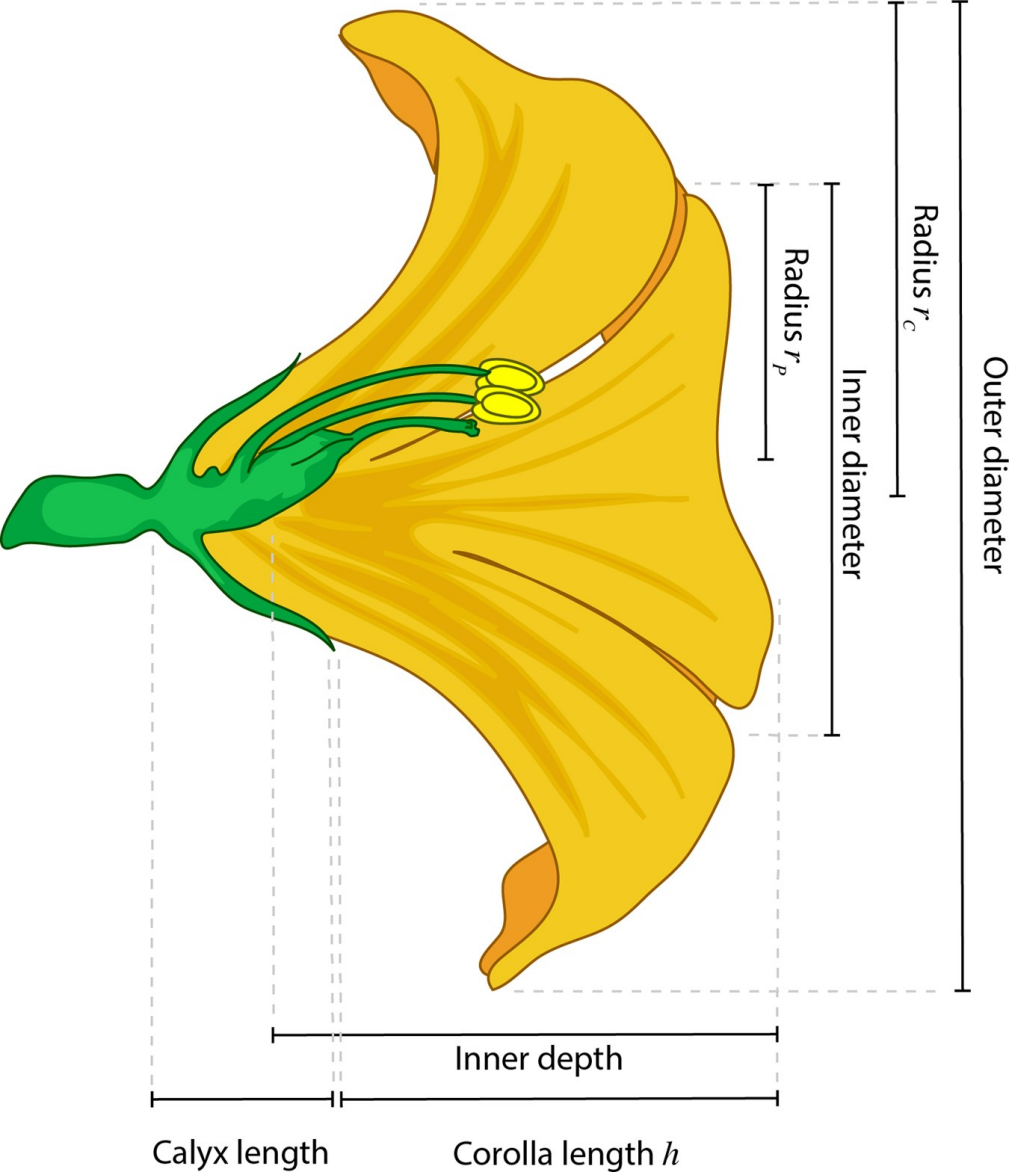
Schematic of the flower morphology measurements. For most of the flowers we took inner and outer diameter of the corolla, inner depth, corolla length and calyx length. If flowers were not axial symmetrical we took corolla diameters in the vertical and in the horizontal plane. The radii (*r*_*p*_ and *r*_*c*_) and corolla length *h* indicated in the schematic were used to estimate flower surface area, see formula (2).

We collected morphological data for each flower including length of the corolla, inner depth, calyx length, inner and outer diameter of the corolla opening for both, the horizontal and vertical axis, see Fig 8. These characteristics were measured with a metal ruler (0.5 mm precision). To estimate the reflective flower surface area *F*_*sur*_, we calculated the area of a bell like form consisting of a circle surface (resembling the corolla opening) and a paraboloid surface (resembling the corolla chalice). We used half of the mean outer corolla diameter as radius *r*_*c*_ to calculate the area of the corolla opening and we used half of the mean inner corolla diameter as radius *r*_*p*_ and the corolla length as height *h* to calculate the outer corolla surface area. The mean corolla diameter was the average of the vertical and horizontal measurements.


$$F_{sur}=\left\{\frac{\pi r_{p}}{6h^{2}}∙[{\left(r_{p}^{2}+4h^{2}\right)}^{\frac{3}{2}}-r_{p}^{3}]\right\}+\pi r_{c}^{2}$$ (2)

As *Witheringia* sp. had a morphology that was very different from the rest of the flowers (Fig 1), we took the following measures for this species: length and width of the petals, length of the calyx, length and width of the anthers. For the species *Condaminea corymbosa* we did not measure calyx length, because it is reduced in this species and hard to measure. As flowers of *Cantua quercifolia* showed radial symmetry we measured inner and outer diameter of the corolla opening only in one plane rather than both horizontal and vertical. For the two latter species we adapted the surface calculation accordingly.

## Supporting information

S1 Fig Dependence of the overall spectral target strength of the 45 kHz frequency band (±20%) on the flower surface area. Bt = *Burmeistera tenuiflora*, Cc = *Condaminea corymbosa*, Cj = *Crescentia cujete*, Cq = *Cantua quercifolia*, Cc = *Centropogon costaricae*, Cs = *Cobaea scandens*, Mm = *Macrocarpea macrophylla*, Mn = *Merinthopodium neuranthum*, Pg = *Palicourea guanensis*, Pp = *Paragonia pyramidata*, Sc = *Symbolanthus calygonus*, Ws = *Witheringia* sp.
(TIF)

S2 Fig Dependence of the overall spectral target strength of the 68 kHz frequency band (±20%) on the flower surface area. Bt = *Burmeistera tenuiflora*, Cc = *Condaminea corymbosa*, Cj = *Crescentia cujete*, Cq = *Cantua quercifolia*, Cc = *Centropogon costaricae*, Cs = *Cobaea scandens*, Mm = *Macrocarpea macrophylla*, Mn = *Merinthopodium neuranthum*, Pg = *Palicourea guanensis*, Pp = *Paragonia pyramidata*, Sc = *Symbolanthus calygonus*, Ws = *Witheringia* sp.
(TIF)

S3 Fig Dependence of the overall spectral target strength of the 102 kHz frequency band (±20%) on the flower surface area. Bt = *Burmeistera tenuiflora*, Cc = *Condaminea corymbosa*, Cj = *Crescentia cujete*, Cq = *Cantua quercifolia*, Cc = *Centropogon costaricae*, Cs = *Cobaea scandens*, Mm = *Macrocarpea macrophylla*, Mn = *Merinthopodium neuranthum*, Pg = *Palicourea guanensis*, Pp = *Paragonia pyramidata*, Sc = *Symbolanthus calygonus*, Ws = *Witheringia* sp.
(TIF)

S4 Fig Dependence of the overall spectral target strength of the 153 kHz frequency band (±20%) on the flower surface area. Bt *= Burmeistera tenuiflora*, Cc *= Condaminea corymbosa*, Cj *= Crescentia cujete*, Cq *= Cantua quercifolia*, Cc *= Centropogon costaricae*, Cs *= Cobaea scandens*, Mm *= Macrocarpea macrophylla*, Mn *= Merinthopodium neuranthum*, Pg *= Palicourea guanensis*, Pp *= Paragonia pyramidata*, Sc *= Symbolanthus calygonus*, Ws *= Witheringia* sp.
(TIF)

## References

[1] Gibson J. The ecological approach to visual perception. Boston: Houghton Mifflin 1979.

[2] Schnitzler H, Moss C, Denzinger A. From spatial orientation to food acquisition in echolocating bats. Trends in Ecology & Evolution. 2003;18(8):386–94.

[3] Winter Y, López J, von Helversen O. Ultraviolet vision in a bat. Nature. 2003;425(6958):612–4. doi: 10.1038/nature01971 14534585

[4] von Helversen O, Winkler L, Bestmann H. Sulphur-containing “perfumes” attract flower-visiting bats. Journal of Comparative Physiology A: Neuroethology, Sensory, Neural, and Behavioral Physiology. 2000;186(2):143–53. doi: 10.1007/s003590050014 10707312

[5] von Helversen D, von Helversen O. Acoustic guide in bat-pollinated flower. Nature. 1999;398(6730):759–60.

[6] von Helversen D, von Helversen O. Object recognition by echolocation: a nectar-feeding bat exploiting the flowers of a rain forest vine. J Comp Physiol A. 2003;189(5):327–36. doi: 10.1007/s00359-003-0405-3 12712362

[7] Simon R, Holderied MW, Koch CU, von Helversen O. Floral acoustics: conspicuous echoes of a dish-shaped leaf attract bat pollinators. Science. 2011;333(6042):631–3. doi: 10.1126/science.1204210 21798950

[8] Gonzalez-Terrazas TP, Koblitz JC, Fleming TH, Medellin RA, Kalko EK, Schnitzler H-U, et al. How nectar-feeding bats localize their food: echolocation behavior of Leptonycteris yerbabuenae approaching cactus flowers. PLoS One. 2016;11(9):e0163492. doi: 10.1371/journal.pone.0163492 27684373 PMC5042408

[9] von Helversen D, Holderied MW, von Helversen O. Echoes of bat-pollinated bell-shaped flowers: conspicuous f or nectar-feeding bats? J Exp Biol. 2003;206(6):1025–34. 12582145 10.1242/jeb.00203

[10] Yovel Y, Stilz P, Franz M, Boonman A, Schnitzler H. What a plant sounds like: The statistics of vegetation echoes as received by echolocating bats. PLoS Comput Biol. 2009;5(7):e1000429. doi: 10.1371/journal.pcbi.1000429 19578430 PMC2699101

[11] Yovel Y, Franz MO, Stilz P, Schnitzler HU. Plant classification from bat-like echolocation signals. PLoS Comput Biol. 2008;4:e1000032. doi: 10.1371/journal.pcbi.1000032 18369425 PMC2267002

[12] Müller R, Kuc R. Foliage echoes: A probe into the ecological acoustics of bat echolocation. J Acoust Soc Am. 2000;108(2):836–45. doi: 10.1121/1.429617 10955651

[13] Emde G, Schnitzler HU. Classification of insects by echolocating greater horseshoe bats. Journal of Comparative Physiology A: Neuroethology, Sensory, Neural, and Behavioral Physiology. 1990;167(3):423–30.

[14] Koselj K, Schnitzler H-U, Siemers BM. Horseshoe bats make adaptive prey-selection decisions, informed by echo cues. Proceedings of the Royal Society B: Biological Sciences. 2011;278(1721):3034–41. doi: 10.1098/rspb.2010.2793 21367788 PMC3158933

[15] Schnitzler H, Menne D, Kober R, Heblich K. IV. 2 The Acoustical Image of Fluttering Insects in Echolocating Bats. Neuroethology and behavioral physiology: roots and growing points. 1983:235.

[16] von Helversen D. Object classification by echolocation in nectar feeding bats: size-independent generalization of shape. J Comp Physiol A. 2004;190(7):515–21. 10.1007/s00359-004-0492-9 15103497

[17] Genzel D, Wiegrebe L. Size does not matter: size-invariant echo-acoustic object classification. Journal of Comparative Physiology A. 2013;199(2):159–68. 10.1007/s00359-012-0777-3 23180047

[18] Firzlaff U, Schuchmann M, Grunwald J, Schuller G, Wiegrebe L. Object-oriented echo perception and cortical representation in echolocating bats. PLoS Biol. 2007;5:1174–83. doi: 10.1371/journal.pbio.0050100 17425407 PMC1847841

[19] Grunwald J, Schörnich S, Wiegrebe L. Classification of natural textures in echolocation. Proceedings of the National Academy of Sciences. 2004;101(15):5670–4. doi: 10.1073/pnas.0308029101 15060282 PMC397469

[20] Schaub A, Schnitzler H. Flight and echolocation behaviour of three vespertilionid bat species while commuting on flyways. Journal of Comparative Physiology A: Neuroethology, Sensory, Neural, and Behavioral Physiology. 2007;193(12):1185–94. doi: 10.1007/s00359-007-0269-z 17885759

[21] Witten IH, Frank E. Data mining: practical machine learning tools and techniques with Java implementations. Acm Sigmod Record. 2002;31(1):76–7.

[22] Thies W, Kalko EKV, Schnitzler HU. The roles of echolocation and olfaction in two Neotropical fruit-eating bats, *Carollia perspicillata* and *C*. *castanea*, feeding on Piper. Behavioral Ecology and Sociobiology. 1998;42(6):397–409.

[23] Kalko EKV, Condon MA. Echolocation, olfaction and fruit display: how bats find fruit of flagellichorous cucurbits. Funct Ecol. 1998;12(3):364–72.

[24] Moss CF, Surlykke A. Auditory scene analysis by echolocation in bats. J Acoust Soc Am. 2001;110:2207–26. doi: 10.1121/1.1398051 11681397

[25] Simmons J, Chen L. The acoustic basis for target discrimination by FM echolocating bats. The Journal of the Acoustical Society of America. 1989;86:1333–50. doi: 10.1121/1.398694 2808908

[26] Schmidt S. Perception of structured phantom targets in the echolocating bat, Megaderma lyra. J Acoust Soc Am. 1992;91:2203–23. doi: 10.1121/1.403654 1597609

[27] Simmons J, Freedman E, Stevenson S, Chen L, Wohlgenant T. Clutter interference and the integration time of echoes in the echolocating bat, Eptesicus fuscus. The Journal of the Acoustical Society of America. 1989;86:1318–32. doi: 10.1121/1.398693 2808907

[28] Simmons J. A view of the world through the bat’s ear: The formation of acoustic images in echolocation. Cognition. 1989;33(1–2):155–99. doi: 10.1016/0010-0277(89)90009-7 2691182

[29] Bradbury J. Target discrimination by the echolocating bat *Vampyrum spectrum*. Journal of Experimental Zoology. 1970;173(1). doi: 10.1002/jez.1401730103 5437462

[30] Simmons J, Lavender W, Lavender B, Doroshow C, Kiefer S, Livingston R, et al. Target structure and echo spectral discrimination by echolocating bats. Science. 1974;186:1130–2. doi: 10.1126/science.186.4169.1130 4469702

[31] Habersetzer J, Vogler B. Discrimination of surface-structured targets by the echolocating bat *Myotis myotis* during flight. Journal of Comparative Physiology A: Neuroethology, Sensory, Neural, and Behavioral Physiology. 1983;152(2):275–82.

[32] Mogdans J, Schnitzler HU, Ostwald J. Discrimination of two-wavefront echoes by the big brown bat, *Eptesicus fuscus*: behavioral experiments and receiver simulations. Journal of Comparative Physiology A: Neuroethology, Sensory, Neural, and Behavioral Physiology. 1993;172(3):309–23. doi: 10.1007/BF00216613 8510056

[33] Simon R, Holderied MW, von Helversen O. Size discrimination of hollow hemispheres by echolocation in a nectar feeding bat. J Exp Biol. 2006;209(18):3599–609. 16943500 10.1242/jeb.02398

[34] Brinkløv S, Kalko EKV, Surlykke A. Intense echolocation calls from two ’whispering’ bats, *Artibeus jamaicensis* and *Macrophyllum macrophyllum* (Phyllostomidae). Journal of Experimental Biology. 2009;212(1):11–20. doi: 10.1242/jeb.023226 19088206

[35] Kuc R. Biomimetic sonar recognizes objects using binaural information. The Journal of the Acoustical Society of America. 1997;102:689–96.

[36] Jones G. Echolocation. Current Biology. 2005;15(13):484–8. 10.1016/j.cub.2005.06.051 16005275

[37] Dobat K, Peikert-Holle T. Blüten und Fledermäuse—Bestaubung durch Fledermäuse und Flughunde (Chiropterophilie). Frankfurt/Main: Waldemar Kramer; 1985.

[38] von Helversen O. Adaptations of flowers to the pollination by glossophagine bats. In: Wea Barthlott, editor. Animal Plant Interactions in Tropical Environments. Bonn: Museum Alexander Koenig; 1993. p. 41–59.

[39] Kroh PK, Simon R, Rupitsch SJ. Classification of Sonar Targets in Air: A Neural Network Approach. Sensors. 2019;19(5):1176. doi: 10.3390/s19051176 30866574 PMC6427766

[40] Simon R, Rupitsch S, Baumann M, Wu H, Peremans H, Steckel J. Bioinspired sonar reflectors as guiding beacons for autonomous navigation. Proceedings of the National Academy of Sciences. 2020;117(3):1367–74. doi: 10.1073/pnas.1909890117 31907314 PMC6983391

[41] Gentry AH. Coevolutionary patterns in central American Bignoniaceae. Annals of the Missouri Botanical Garden. 1974;61(3):728–59.

[42] Muchhala N. Exploring the boundary between pollination syndromes: bats and hummingbirds as pollinators of Burmeistera cyclostigmata and B. tenuiflora (Campanulaceae). Oecologia. 2003;134(3):373–80. doi: 10.1007/s00442-002-1132-0 12647145

[43] Temeles EJ, Linhart YB, Masonjones M, Masonjones HD. The Role of Flower Width in Hummingbird Bill Length-Flower Length Relationships 1. Biotropica. 2002;34(1):68–80.

[44] Vogel S. Fledermausblumen in Südamerika. Plant Systematics and Evolution. 1958;104(4):491–530.

[45] Knudsen JT, Tollsten L, Groth I, Bergström G, Raguso RA. Trends in floral scent chemistry in pollination syndromes: floral scent composition in hummingbird-pollinated taxa. Botanical journal of the Linnean Society. 2004;146(2):191–9.

[46] Grant KA. A hypothesis concerning the prevalence of red coloration in California hummingbird flowers. The American Naturalist. 1966;100(911):85–97.

[47] Taylor CM. Conspectus of the genus Palicourea (Rubiaceae: Psychotrieae) with the description of some new species from Ecuador and Colombia. Annals of the Missouri Botanical Garden. 1997:224–62.

[48] Tschapka M. Energy density patterns of nectar resources permit coexistence within a guild of Neotropical flower-visiting bats. Journal of Zoology. 2004;263(01):7–21.

[49] Bohs L. Insights into the Witheringia solanacea (Solanaceae) Complex in Costa Rica. I. Breeding Systems and Crossing Studies 1. Biotropica. 2000;32(1):70–9.

[50] Dmitrieva M, Valdenegro-Toro M, Brown K, Heald G, Lane D, editors. Object classification with convolution neural network based on the time-frequency representation of their echo. 2017 IEEE 27th International Workshop on Machine Learning for Signal Processing (MLSP); 2017: IEEE.

[51] Sun Y, Zhu L, Wang G, Zhao F. Multi-input convolutional neural network for flower grading. Journal of Electrical and Computer Engineering. 2017;2017.

[52] Kingma DP, Ba J. Adam: A method for stochastic optimization. arXiv preprint arXiv:14126980. 2014.

