# Supplementary figures and images for: Acoustic traits of bat-pollinated flowers compared to flowers of other pollination syndromes and their echo-based classification using convolutional neural networks
Source: PLoS Comput Biol. 2021 Dec 16;17(12):e1009706. doi: 10.1371/journal.pcbi.1009706 (PMC8718002; doi:10.1371/journal.pcbi.1009706)

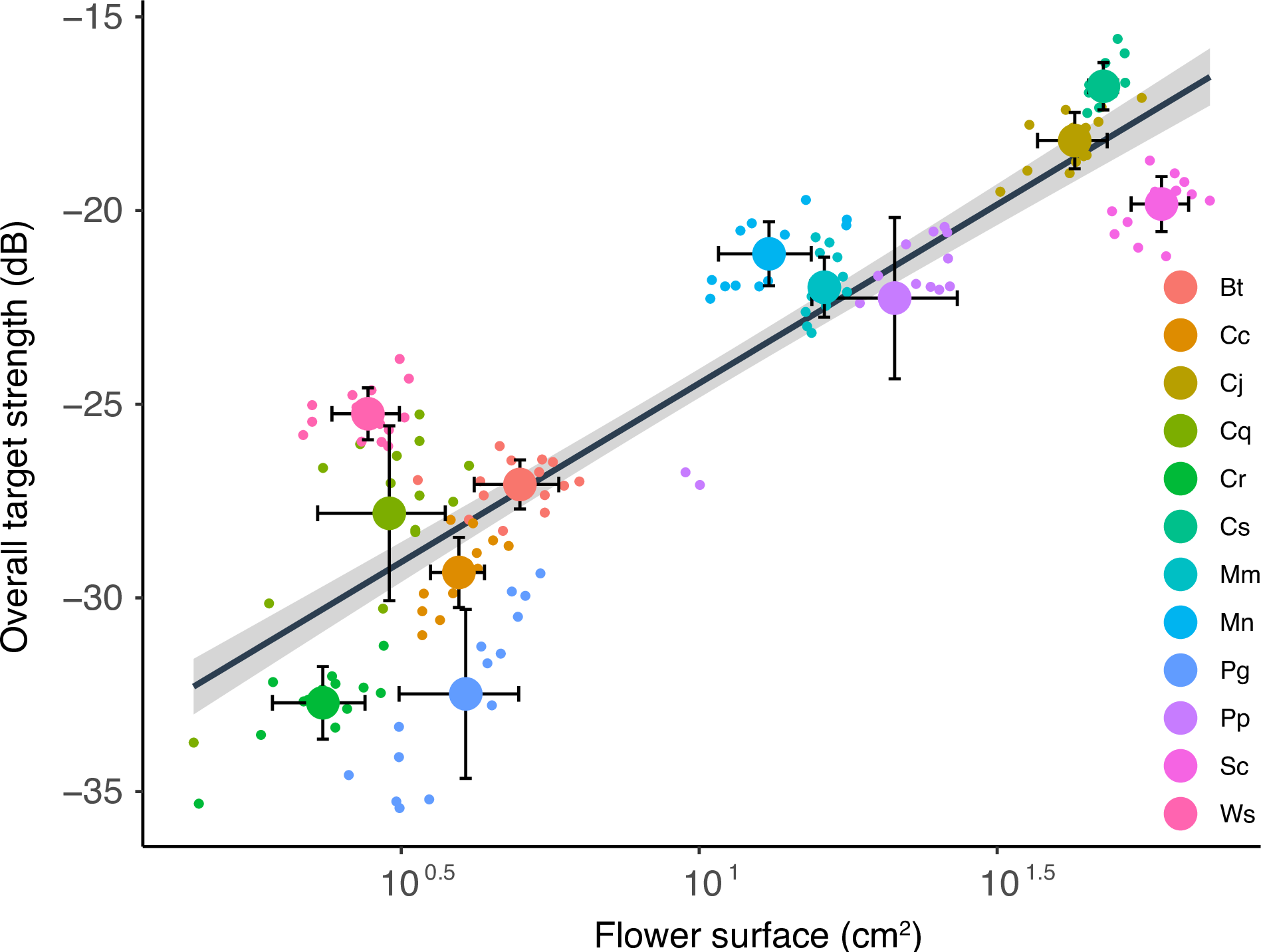

Supplement: S1 Fig — Bt = Burmeistera tenuiflora, Cc = Condaminea corymbosa, Cj = Crescentia cujete, Cq = Cantua quercifolia, Cc = Centropogon costaricae, Cs = Cobaea scandens, Mm = Macrocarpea macrophylla, Mn = Merinthopodium neuranthum, Pg = Palicourea guanensis, Pp = Paragonia pyramidata, Sc = Symbolanthus calygonus, Ws = Witheringia sp. (TIF) [file pcbi.1009706.s001.tif]

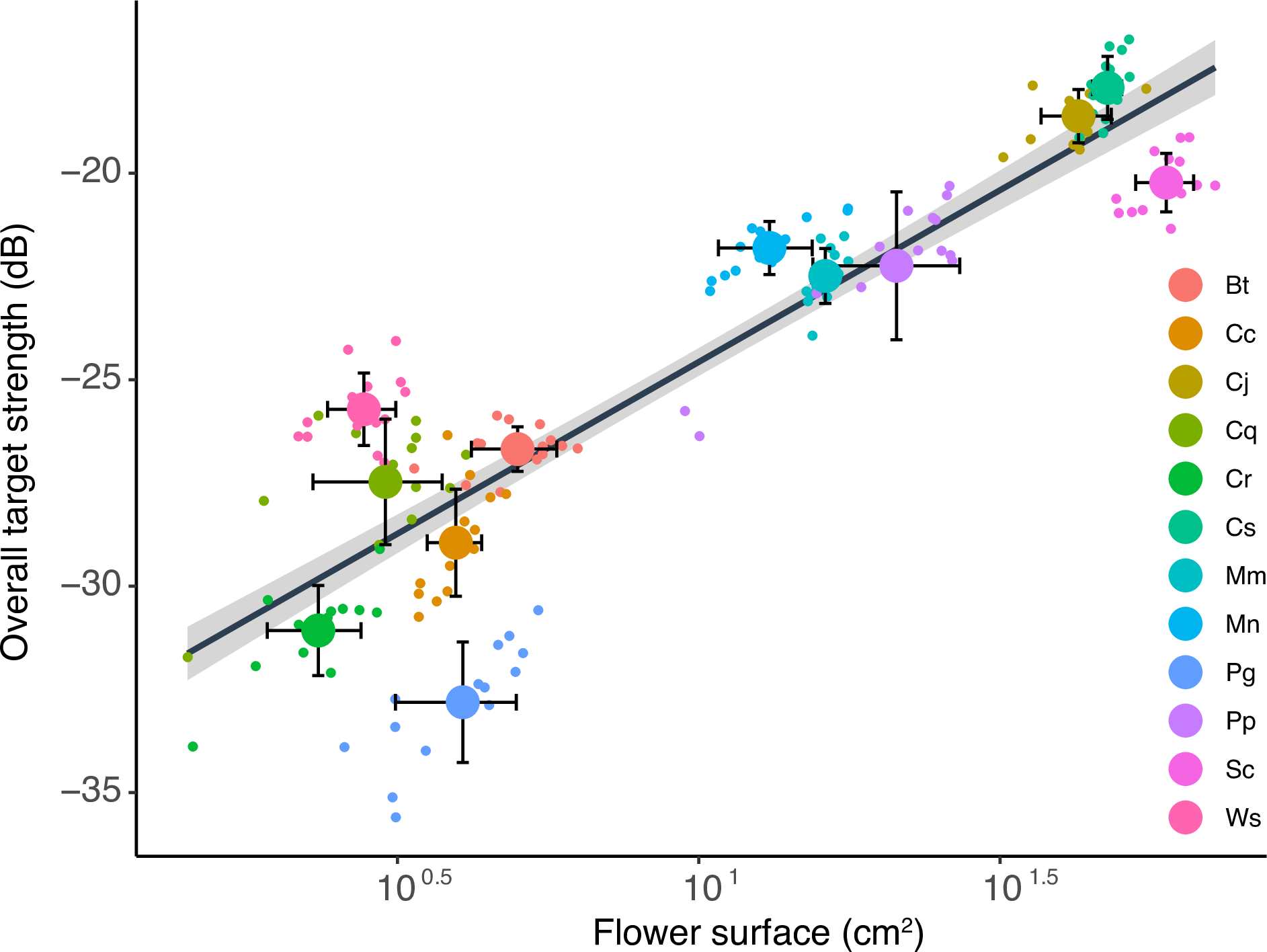

Supplement: S2 Fig — Bt = Burmeistera tenuiflora, Cc = Condaminea corymbosa, Cj = Crescentia cujete, Cq = Cantua quercifolia, Cc = Centropogon costaricae, Cs = Cobaea scandens, Mm = Macrocarpea macrophylla, Mn = Merinthopodium neuranthum, Pg = Palicourea guanensis, Pp = Paragonia pyramidata, Sc = Symbolanthus calygonus, Ws = Witheringia sp. (TIF) [file pcbi.1009706.s002.tif]

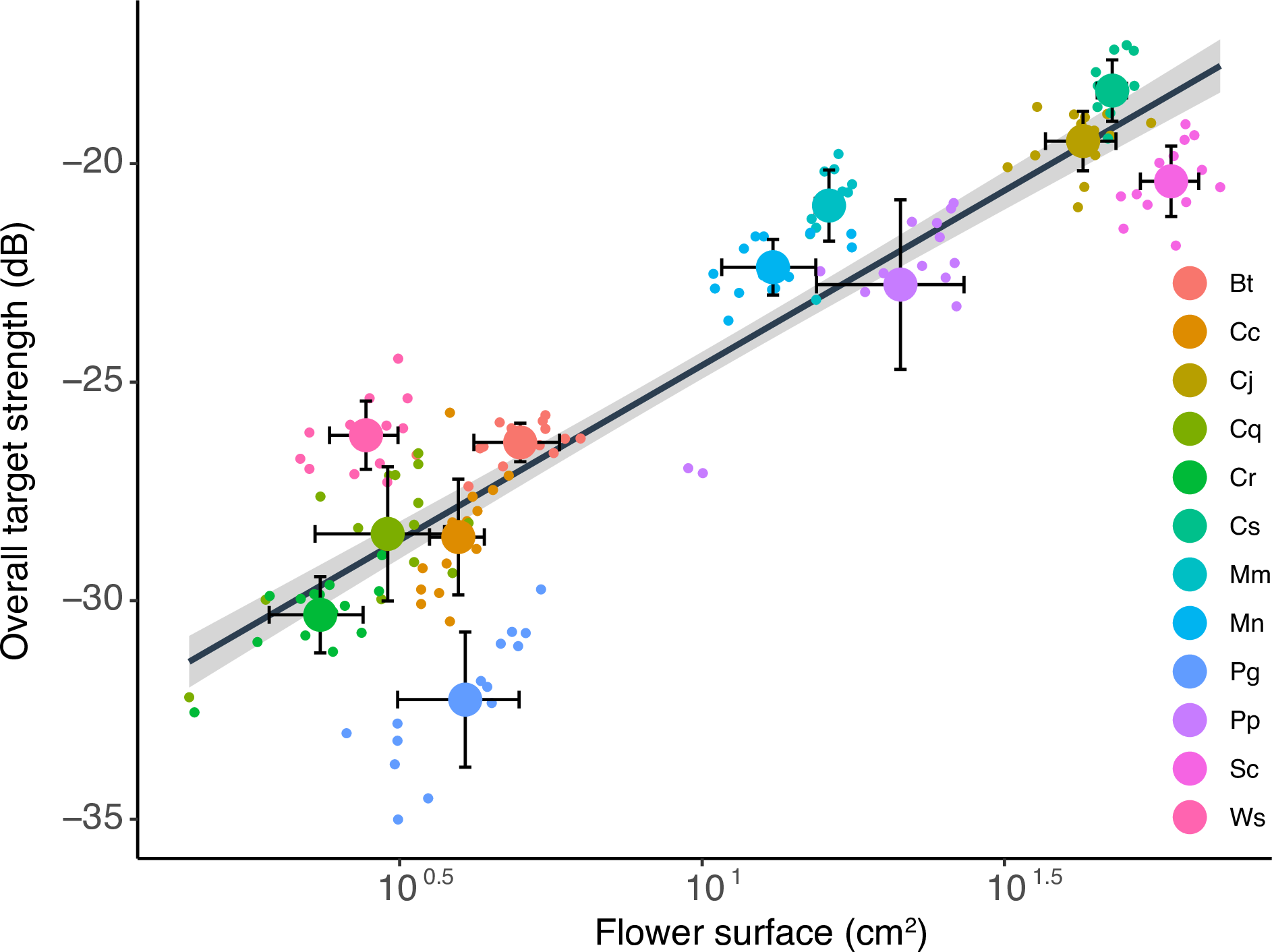

Supplement: S3 Fig — Bt = Burmeistera tenuiflora, Cc = Condaminea corymbosa, Cj = Crescentia cujete, Cq = Cantua quercifolia, Cc = Centropogon costaricae, Cs = Cobaea scandens, Mm = Macrocarpea macrophylla, Mn = Merinthopodium neuranthum, Pg = Palicourea guanensis, Pp = Paragonia pyramidata, Sc = Symbolanthus calygonus, Ws = Witheringia sp. (TIF) [file pcbi.1009706.s003.tif]

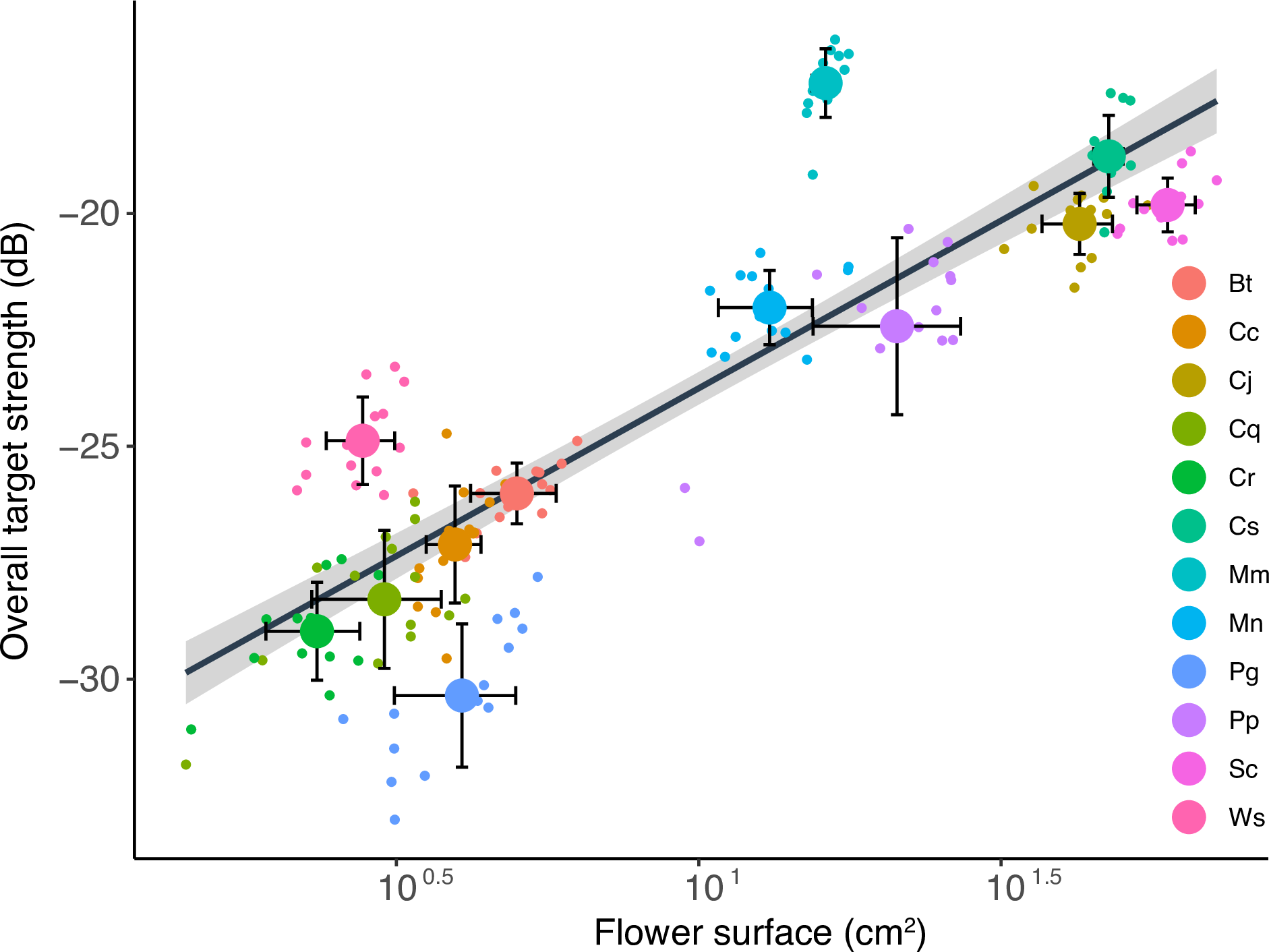

Supplement: S4 Fig — Bt = Burmeistera tenuiflora, Cc = Condaminea corymbosa, Cj = Crescentia cujete, Cq = Cantua quercifolia, Cc = Centropogon costaricae, Cs = Cobaea scandens, Mm = Macrocarpea macrophylla, Mn = Merinthopodium neuranthum, Pg = Palicourea guanensis, Pp = Paragonia pyramidata, Sc = Symbolanthus calygonus, Ws = Witheringia sp. (TIF) [file pcbi.1009706.s004.tif]
